# The *Streptomyces coelicolor* Small ORF *trpM* Stimulates Growth and Morphological Development and Exerts Opposite Effects on Actinorhodin and Calcium-Dependent Antibiotic Production

**DOI:** 10.3389/fmicb.2020.00224

**Published:** 2020-02-19

**Authors:** Alberto Vassallo, Emilia Palazzotto, Giovanni Renzone, Luigi Botta, Teresa Faddetta, Andrea Scaloni, Anna Maria Puglia, Giuseppe Gallo

**Affiliations:** ^1^Laboratory of Molecular Microbiology and Biotechnology, STEBICEF Department, University of Palermo, Palermo, Italy; ^2^Laboratory of Microbial and Molecular Evolution, Department of Biology, University of Florence, Sesto Fiorentino, Italy; ^3^Novo Nordisk Foundation Center for Biosustainability, Technical University of Denmark, Lyngby, Denmark; ^4^Proteomic and Mass Spectrometry Laboratory, ISPAAM, National Research Council, Naples, Italy; ^5^Dipartimento di Ingegneria, Università di Palermo, Palermo, Italy

**Keywords:** small open reading frame, *trpM*, actinorhodin production, *Streptomyces coelicolor*, cytosol aminopeptidase, calcium-dependent antibiotic, primary and secondary metabolism

## Abstract

In actinomycetes, antibiotic production is often associated with a morpho-physiological differentiation program that is regulated by complex molecular and metabolic networks. Many aspects of these regulatory circuits have been already elucidated and many others still deserve further investigations. In this regard, the possible role of many small open reading frames (smORFs) in actinomycete morpho-physiological differentiation is still elusive. In *Streptomyces coelicolor*, inactivation of the smORF *trpM* (SCO2038) – whose product modulates L-tryptophan biosynthesis – impairs production of antibiotics and morphological differentiation. Indeed, it was demonstrated that TrpM is able to interact with PepA (SCO2179), a putative cytosol aminopeptidase playing a key role in antibiotic production and sporulation. In this work, a *S. coelicolor trpM* knock-in (Sco-*trpM*KI) mutant strain was generated by cloning *trpM* into overexpressing vector to further investigate the role of *trpM* in actinomycete growth and morpho-physiological differentiation. Results highlighted that *trpM*: (i) stimulates growth and actinorhodin (ACT) production; (ii) decreases calcium-dependent antibiotic (CDA) production; (iii) has no effect on undecylprodigiosin production. Metabolic pathways influenced by *trpM* knock-in were investigated by combining two-difference in gel electrophoresis/nanoliquid chromatography coupled to electrospray linear ion trap tandem mass spectrometry (2D-DIGE/nanoLC-ESI-LIT-MS/MS) and by LC-ESI-MS/MS procedures, respectively. These analyses demonstrated that over-expression of *trpM* causes an over-representation of factors involved in protein synthesis and nucleotide metabolism as well as a down-representation of proteins involved in central carbon and amino acid metabolism. At the metabolic level, this corresponded to a differential accumulation pattern of different amino acids – including aromatic ones but tryptophan – and central carbon intermediates. PepA was also down-represented in Sco-*trpM*KI. The latter was produced as recombinant His-tagged protein and was originally proven having the predicted aminopeptidase activity. Altogether, these results highlight the stimulatory effect of *trpM* in *S. coelicolor* growth and ACT biosynthesis, which are elicited through the modulation of various metabolic pathways and PepA representation, further confirming the complexity of regulatory networks that control antibiotic production in actinomycetes.

## Introduction

Bacteria belonging to the *phylum Actinomycetales* are widely recognized as a very prolific source of biologically active natural compounds, such as antibiotics, immunosuppressants, and herbicides. As reviewed by Palazzotto et al. (2019), different approaches have been adopted to increase production of these molecules in actinomycetes, ranging from metabolic engineering strategies – driving the nutrient catabolism toward an increased supply of precursors – to the modification of the expression of regulators taking directly part in natural product biosynthesis. Besides being studied as a model for investigation of bacterial differentiation, the filamentous Gram-positive bacterium *Streptomyces coelicolor* is a model organism for the study of polyketide antibiotic production in *Actinomycetales*. *S. coelicolor* produces different biologically active metabolites whose biosynthesis has been widely documented – including the blue-pigmented Type II polyketide actinorhodin (ACT), the red-pigmented alkaloid undecylprodigiosin (RED), and the cyclic lipoundecapeptide calcium-dependent antibiotic (CDA) (Liu et al., 2013). In *S. coelicolor*, many aspects of regulatory circuits controlling antibiotic production have been already elucidated although many others still deserve further investigations. As an example, Xu et al. (2019) demonstrated that ACT biosynthesis is affected by a number of genes spread along the chromosome that have been never previously associated with production of ACT. Also, supplementation of specific nutriments exerts a control on *S. coelicolor* morphological and physiological differentiation: as an example, Palazzotto et al. (2015) demonstrated that the supplementation of L-tryptophan (L-Trp) promotes sporulation and stimulates the production of CDA – that contains proteinogenic and non-proteinogenic amino acids, including L-Trp and D-Trp – and the production of ACT – that does not contain any Trp in its structure. In this regard, it is noteworthy that in *S. coelicolor* the expression of genes involved in L-Trp biosynthesis (i.e., *trp* genes) is not repressed by Trp supplementation (Hu et al., 1999; Palazzotto et al., 2016). In addition, *trp* genes are organized either as gene clusters (i.e., *trpC1MBA* and *trpC2D2GE2*) or single genes (i.e., *trpE3*, *trpE1* and *priA*/*trpF*) spread in the genome with *trpC2D2GE2* localized within the CDA biosynthetic gene cluster. So, this gene organization probably allows *S. coelicolor* to express a subset of *trp* genes independently from the others and in response to specific metabolic needs (Xie et al., 2003).

*trpM* (SCO2038) is part of the *trpC1MBA locus* (Hu et al., 1999; Palazzotto et al., 2016). Because of its small size (just 64 codons), *trpM* and the corresponding protein can be listed as a small open reading frame (smORF) and a small open reading frame-encoded protein (SEP), respectively. smORFs and SEPs have been extensively ignored so far and discovered mostly serendipitously. Nonetheless, they are known to take part in different important cell processes in bacteria – e.g., spore formation, cell division, membrane transport, regulation of enzymatic activities and signal transduction – and nowadays they are gaining more and more attention (Storz et al., 2014; Chu et al., 2015; Chugunova et al., 2018; Delcourt et al., 2018). It was previously demonstrated that TrpM is involved in L-Trp biosynthesis (Palazzotto et al., 2016). Indeed, a *trpM*-knockout mutant strain showed an impaired growth on minimal medium but a normal growth was restored upon addition of L-Trp or its precursors (i.e., L-serine and indole) to the medium. These results were corroborated through a proteomic investigation that compared the *trpM*-knockout mutant and wild type strains, showing that the list of differentially abundant proteins included some components directly involved in L-Trp biosynthesis (Palazzotto et al., 2016). Moreover, the inactivation of *trpM* significantly affected ACT biosynthesis, since the *trpM*-knockout mutant produced 10-fold less ACT than the WT strain. In addition, a pull-down assay using immobilized His-tagged TrpM allowed to identify the putative cytosol L-leucine-aminopeptidase PepA belonging to M17 metalloprotease family (SCO2179), the ribosomal protein S1 (SCO1998) and the ribosomal protein S2 (SCO5624) as probable interacting proteins of this SEP. TrpM and PepA interaction was also demonstrated by bacterial two-hybrid assay (Palazzotto et al., 2016).

In this work, a *trpM* knock-in mutant strain was obtained to characterize the role of TrpM in both morphological differentiation and antibiotic biosynthesis of *S. coelicolor*; results from different comparative experiments on this mutant and wild type strains allowed us to suggest a possible molecular model explaining its mode of action.

## Materials and Methods

### Bacterial Strains, Plasmids, and Cultivation Conditions

All *Streptomyces coelicolor* strains and plasmids used in this work are listed in Table 1. Besides, *Escherichia coli* TOP10 (Invitrogen), *E. coli* S17-1 (Simon et al., 1983), and *E. coli* BL21-AI (Invitrogen) were used as described below.

**TABLE 1:** List of strains and plasmids used in this work.

Strain/plasmid	Genotype	References
*S. coelicolor* M145	SCP1^–^ SCP2^–^	Kieser et al., 2000
*S. coelicolor* M145 carrying pIJ8600 (Sco-EV)	SCP1^–^ SCP2^–^*attBΦC31*: pIJ8600	This work
*S. coelicolor* M145 carrying pIJ8600:*trpM* (Sco-*trpM*KI)	SCP1^–^ SCP2^–^*attBΦC31*: pIJ8600:*trpM*	This work

pIJ8600	AprR, TsrR	Sun et al., 1999
pIJ8600:*trpM*	AprR, TsrR	This work
pRSET-B	AmpR	Thermo Fisher Scientific
pRSET-B:*pepA*	AmpR	This work

The *Escherichia coli* strains were cultivated in LB medium (Sambrook and Russell, 2001) supplemented with apramycin (50 μg/mL) and ampicillin (100 μg/mL) in the case of strains carrying pIJ8600/pIJ8600:*trpM* and pRSET-B/pRSET-B:*pepA*, respectively, at 37°C and 200 rpm. For *S. coelicolor* cultures, minimal medium [NaNO_3_ (1 g/L), MgSO_4_7H_2_O (0.5 g/L), KCl (0.5 g/L), KH_2_PO_4_ (1 g/L), glucose (10 g/L), trace element solution (1 mL/L), pH 7 as adjusted before sterilization] was used; trace element solution contained FeSO_4_7H_2_O (1 g/100 mL), ZnCl_2_ (1 g/100 mL), and biotin (0.1 g/100 mL). Glucose and trace element solution were added upon sterilization, and solid media were prepared adding bacto agar (15 g/L) to the recipes reported. If not otherwise indicated, 1.5 × 10^7^ spores of *S. coelicolor* strains were spread on solid minimal medium, and incubated at 30°C, for 7 days. In the case of cultures used for RNA, protein and metabolite extraction, a disc of cellophane (Cellophane Membrane Backing, Bio-Rad, United States) was placed on the surface of medium to facilitate mycelium harvesting.

### Construction of Recombinant *Streptomyces coelicolor* Strains

DNA manipulation, purification, ligation, restriction analysis, gel electrophoresis and transformation of *E. coli* were performed according to standard techniques (Sambrook and Russell, 2001). *trpM* and *pepA* were amplified from genomic DNA of *S. coelicolor* M145 using the couple of primers trpM_exp_F/trpM_exp_R and pepA_exp_F/pepA_exp_R, respectively, which are reported in Table 2. *Taq* DNA Polymerase Recombinant (Invitrogen) was used in both cases, and standard conditions indicated by the manufacturer were adopted. Purified PCR products containing *trpM* and *pepA* genes were digested with restriction enzymes (*Nde*I and *Bam*HI in case of *trpM*, while *Bam*HI and *Hin*dIII were used for *pepA*), and ligated into previously restricted pIJ8600 and pRSET-B vectors, respectively (Tables 1, 2). Ligation mix was then used for transformation of One Shot Chemically Competent *E. coli* TOP10 cells (Invitrogen). The identity of all DNA fragments amplified by PCR was confirmed by DNA sequencing. The pIJ8600 and pIJ8600:*trpM* plasmids were used to transform chemically competent *E. coli* S17-1 cells, which were used as donor ones to transform *S. coelicolor* M145 through a conjugation-based protocol (Kieser et al., 2000). Integration of either pIJ8600 and pIJ8600:*trpM* plasmids in the *attBΦC31* site of the chromosomal DNA of *S. coelicolor* was verified through Southern blotting. The 1480 bp-long restriction fragment derived from digestion of pIJ8600 with *Sty*I was labeled using the Dig High Prime DNA Labeling and Detection Starter kit I (Roche), and used as probe. Chromosomal DNA of *S. coelicolor* strains was digested with *Bam*HI and blotting was performed according to the manufacturer’s instructions and Sambrook and Russell (2001).

**TABLE 2:** List of primers used in this work.

Primer	Sequence (5′ > 3′)*	Application
trpM_exp_F	CGGACATATGATGACGCTCC	Construction of
trpM_exp_R	CGGGGATCCTCAATACAGC	pIJ8600:*trpM*
pepA_exp_F	ATAAGGATCCGTGACTGCTC	Construction of
pepA_exp_R	GATCAAGCTTCTAGCCCAG	pRSET-B:*pepA*
hrdB_F	GGTCGAGGTCATCAACAAGC	qRT-PCR (*hrdB*)
hrdB_R	CTCGATGAGGTCACCGAACT	
SCO2038_F	CGCTCCCGCTCGTCCC	qRT-PCR (*trpM*)
SCO2038_R	CCTGATGGGGCGCTTGA	
SCO2179_F	CGCCCAGGCCGTGGACA	qRT-PCR (*pepA*)
SCO2179_R	CCACGACGACGGGAGCCT	

*Underlined sequence corresponds to restriction sites.

### Transcriptional Analysis

RNA was extracted using the Illustra RNAspin Midi RNA Isolation Kit (GE Healthcare, United States) and according to the protocol provided by the manufacturer. A two-step protocol was applied for qRT-PCR and the High-Capacity cDNA Reverse Transcription Kit with RNase Inhibitor (Applied Biosystems) was firstly used to convert RNA in cDNA. Thus, Power SYBR Green PCR Master Mix (Applied Biosystems) was used for relative RNA quantification. To this purpose, primers reported in Table 2 were used, and *hrdB* was chosen as internal standard. Two biological replicates and three technical replicates were used for each condition.

### Scanning Electron Microscope Observations

Agar block samples (1 cm × 1 cm × 0.5 cm) were cut from agar-medium cultures, washed three times with phosphate-buffered saline (PBS) for 3 min, and then fixed using 4% v/v glutaraldehyde for 5 min, under gentle agitation. Upon removing of glutaraldehyde solution, samples were washed with 15% v/v ethanol for 3 min, and incubated at 65°C until obtaining a thin slice. Samples were sputter-coated with gold to avoid electrostatic charging under the electron beam and examined by Scanning Electronic Microscopy (SEM) (Phenom ProX, PhenomWorld).

### Bacterial Growth Kinetics

An amount of ≃10^8^ spores of *S. coelicolor* M145 strains carrying pIJ8600:*trpM* (Sco-*trpM*KI) and pIJ8600 (Sco-EV) were inoculated in 25 mL of J medium (Kieser et al., 2000), respectively, using 250 mL baffled flasks, and incubated for 30 h (30°C, 200 rpm in an orbital shaker). Cultures were centrifuged at 3000 × *g* for 15 min, and pellets were washed twice with sterile water. Finally, pellets were resuspended in 50 mL of sterile water, and 3 mL of the obtained suspensions were inoculated in 200 mL of minimal medium (MM), using 1 L baffled flasks, and incubated for 4 days (30°C, 200 rpm in an orbital shaker). Every 12 h, three aliquots of 1 mL were sampled for dry weight determination. Thus, they were centrifuged, decanted, dried at 65°C for 24 h, and finally weighed.

### Spore Counting and Antibiotic Assays

An amount of ≃1.5 × 10^7^ spores of Sco-*trpM*KI and Sco-EV were spread on six MM agar plates, and incubated at 30°C, for 7 days. Three of them were used for spore harvesting and a subsequent serial dilution counting, while the others were used for determination of ACT and undecylprodigiosin production. Antibiotic assays were performed as described by Kieser et al. (2000). In particular, total ACT and undecylprodigiosin (RED) were sequentially extracted by treating cultivations with a 1 N KOH aqueous solution and a 0.5 N HCl/methanol solution, respectively. Insoluble matter, resulting as pellet after 1 N KOH treatment and centrifugation (13000 x *g*, for 15 min), was washed twice with 1 M Tris–HCl, pH 7.5, before treatment with a solution of 0.5 N HCl/methanol 50:50 v/v (Scaffaro et al., 2017). The amount of ACT and RED, whose identity was confirmed by visible absorption spectra (Horinouchi and Beppu, 1984), was spectrophotometrically evaluated at 640 and 530 nm as the mean value measured for three independent cultivations. Concentrations of ACT and RED were determined using molar extinction coefficients of pure compounds. They are ε_640_ = 25320 M^–1^ x cm^–1^ and ε_530_ = 100500 M^–1^ x cm^–1^ for ACT and RED, respectively. CDA production was evaluated according to Kieser et al. (2000) and Palazzotto et al. (2015).

### Proteomic Analysis

Proteins were extracted from the mycelium of Sco-*trpM*KI and Sco-EV according to Puglia et al. (1995), and proteomic investigation was conducted by 2D-DIGE as described by Palazzotto et al. (2016), except that 1.3 was chosen as threshold value for the identification of differentially represented proteins. ANOVA test was used to assess the statistical significance of protein abundance fold change, using a *p* ≤ 0.05 to consider differentially represented proteins. Proteins were extracted from three biological replicas for each strain and 2D-DIGE procedure was conducted with two technical replicas with a total of six replicas *per* strain. Identification of protein material present in each differentially represented spot was performed by nanoLC-ESI-LIT-MS/MS analysis, which was performed with an LTQ XL mass spectrometer (Thermo Fisher Scientific, United States) equipped with a Proxeon nanospray source connected to an Easy nanoLC (Thermo Fisher Scientific, United States) (Lirussi et al., 2012). Peptide mixtures were resolved on an Easy C18 column (10 – 0.075 mm, 3 μm) (Thermo Fisher Scientific, United States) as previously reported (Palazzotto et al., 2016).

MASCOT search engine version 2.2.06 (Matrix Science, United Kingdom) was used to identify protein spots from an updated NCBI non-redundant database (downloaded January 2018) also containing protein sequences for *S. coelicolor* A3(2), using mass spectrometric data. Database searching was performed selecting trypsin as proteolytic enzyme, a missed cleavages maximum value of 2, Cys carbamidomethylation as fixed modification, Met oxidation and N-terminal Gln conversion to pyro-Glu as variable modifications, respectively. Candidates with at least 2 assigned peptides with an individual peptide expectation value less than 0.05, which corresponds to a confidence level for peptide attribution greater than 95%, were further evaluated by the comparison with their calculated mass and p*I* values, using the experimental data obtained from electrophoresis. Protein assignment was always associated with manual verification. Finally, in case of multiple protein identifications, unambiguous protein identity was assigned according to an emPAI ratio criterion calculated between the two most abundant protein species (i.e., emPAI 1^st^/emPAI 2^nd^ > 1.50) (Shinoda et al., 2010). The mass spectrometry proteomics data have been deposited to the ProteomeXchange Consortium via the PRIDE (Perez-Riverol et al., 2019) partner repository with the dataset identifier PXD015937.

### Metabolic Profile Determination

Metabolic profiles of Sco-*trpM*KI and Sco-EV were investigated through LC-ESI-MS/MS analysis in multiple reaction monitoring (MRM) mode coupled with protein precipitation and extraction of metabolites using organic solvents. All procedures reported below were carried out at Centro di Ingegneria Genetica (CEINGE) – Biotecnologie avanzate (Naples, Italy). Samples were lysed in 250 μL of a buffer containing 10 mM NH_4_CO_3_, 10 mM NaF, 7 M urea, 75 mM NaCl, and the suspension thus obtained was homogenized. Cellular lysates were fivefold diluted with a cold solution of acetonitrile/methanol (50:50 v/v) containing 0.1% v/v acetic acid. Samples were sonicated for 10 min, and centrifuged at 12000 rpm for 10 min, at 4°C. Supernatants were dried and resuspended in 500 μL of a solution of acetonitrile/methanol/water (40:40:20 v/v/v) containing 0.1% v/v acetic acid. Upon centrifugation at 12000 rpm for 10 min, supernatants were filtered using 0.22 μm centrifugal filters and analyzed by LC-ESI-MS/MS. Quantitative analysis was performed by the external calibration method. Standard solutions were prepared dissolving 1 mg of each analyte in 1 mL of 5% v/v acetonitrile (1000 ppm), and serial dilutions of them were used for the generation of calibration curves. 1 μL of supernatants obtained from samples were analyzed with a 6420 Triple Quadrupole System (SCIEX, United States) coupled to a HPLC 1100 Series Binary Pump (Agilent, United States). Analytes were separated using a Kinetex 5 μm C18 analytical column (100 mm × 2.1 mm) (Phenomenex). The mobile phase was generated by mixing eluent A (0.1% v/v acetic acid containing 3 mM ammonium acetate) and eluent B (50% v/v acetonitrile, 50% v/v 2-propanol and 0.1% v/v acetic acid), at a flow rate of 0.3 mL/min. The elution gradient ranged from 5% to 95% of eluent B in 7 min. Tandem mass spectrometry was performed using a turbo ion spray source operating in negative mode and MRM mode was used for the selected analytes. Metabolites were tuned for ionization polarity, optimal declustering potential, precursor and daughter product ions, and collision energy. Metabolites were extracted from two biological replicates of both strains and three technical replicates of each one were processed. Student’s *t*-test was applied to verify the statistical significance of metabolite abundance fold change, using a *p* ≤ 0.05 to consider differentially represented metabolites.

### Over-Expression of PepA in *Escherichia coli*

One Shot Chemically Competent *E. coli* BL21-AI cells (Invitrogen) were transformed with either pRSET-B:*pepA* or pRSET-B, with the former used to obtain a 6xHis-tagged recombinant PepA and the latter to have a control protein extract. In particular, an amount of 50 mL of LB medium was inoculated with 2.5 mL of an overnight-grown culture of *E. coli* BL21-AI carrying pRSET-B:*pepA*. The culture was incubated (37°C, at 200 rpm in an orbital shaker) until OD_600_ reached a value of 0.8. Then, a sterile solution of the inducer L-arabinose was added to have a final concentration of 0.1% w/v, and induction of *pepA* over-expression was performed at 30°C, for 3 h. *E. coli* BL21-AI carrying pRSET-B was used as negative control, and aliquots from all cultures were collected as further control before adding the inducer. Cultures were centrifuged, and the pellet resuspended in 2 mL of 50 mM Tris–HCl, pH 8. Then, suspensions were sonicated with 3 pulses (10 sec each and 4 as output control) in an ice bath and centrifuged (30 min, 7000 × *g*, at 4°C). Supernatants containing the water-soluble (WS) protein fraction of the cell lysate were directly used for leucine aminopeptidase assay measurements. Bradford reagent was used to determine WS protein concentration (Bradford, 1976).

### Leucine Aminopeptidase Assay

An aliquot of 400 μg of WS proteins was added to the reaction mix (0.25 mM L-leucine-*p*-nitroanilide, 1 mM CoCl_2_ or MnCl_2_, 50 mM Tris–HCl, pH 8) in a final volume of 500 μL. After incubation at 60°C for 10 min, a volume of 10% v/v acetic acid was added, and the solution was incubated at 100°C, for 5 min. Samples were finally centrifuged at 13000 × *g*, for 5 min, at 4°C, and the absorbance of supernatants was measured at 405 nm (Kuo et al., 2003; Song et al., 2013). This assay was performed with three different technical replicates.

## Results

### Construction and Characterization of a *trpM* Knock-in Mutant Strain

For the construction of a *S. coelicolor trpM* knock-in mutant strain (thereof named Sco-*trpM*KI), *trpM* was PCR-amplified and cloned within the multiple cloning site of the integrative expression vector pIJ8600 under the control of the thiostrepton-inducible promoter (P*tipA*) (Murakami et al., 1989; Kieser et al., 2000). This plasmid was delivered to *S. coelicolor* through interspecific conjugation, and the correct integration in the *attB* ΦC31 site was verified by PCR, sequencing and Southern blotting. Analogously, a strain having the empty vector pIJ8600 integrated in its genome (indicated as Sco-EV) was constructed and used as control during all the following experiments. Since qRT-PCR showed that the expression of *trpM* in Sco-*trpM*KI was already twofold greater than that in Sco-EV (Supplementary Figure S1), thiostrepton was not added to the growth medium as inducer to avoid the introduction of any possible negative effects on antibiotic production and on protein expression pattern as described by Wang et al. (2017) and Chiu et al. (1999), respectively.

**FIGURE 1:**
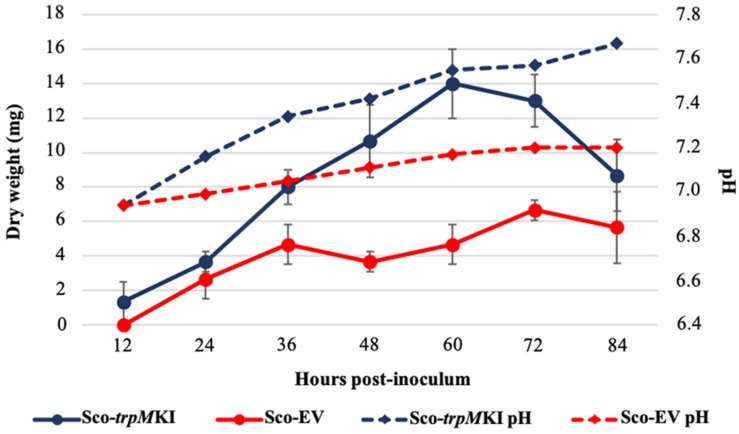
Growth kinetics (solid lines) and pH profile (dashed lines) of Sco-*trpM*KI (blue lines) and Sco-EV (red lines) cultures performed in liquid MM.

**FIGURE 2:**
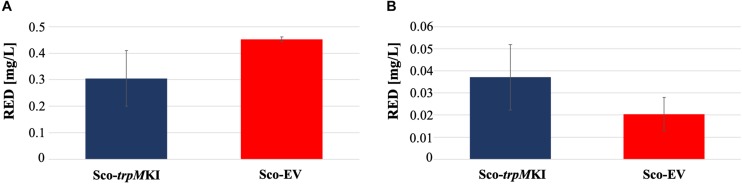
Undecyilprodigiosin (RED) production in cultures of Sco-*trpM*KI and Sco-EV strains performed using liquid **(A)** and solid **(B)** MM growth-medium.

**FIGURE 3:**
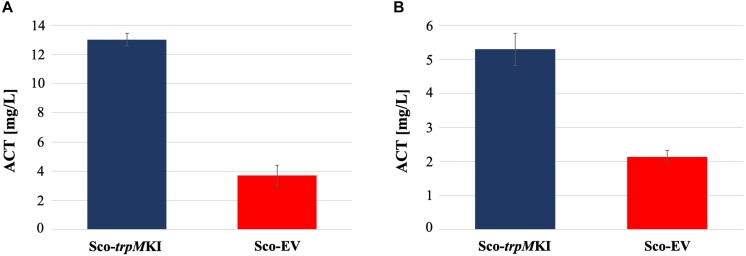
Actinorhodin (ACT) production in cultures of Sco-*trpM*KI and Sco-EV strains performed using liquid **(A)** and solid **(B)** MM growth-medium.

**FIGURE 4:**
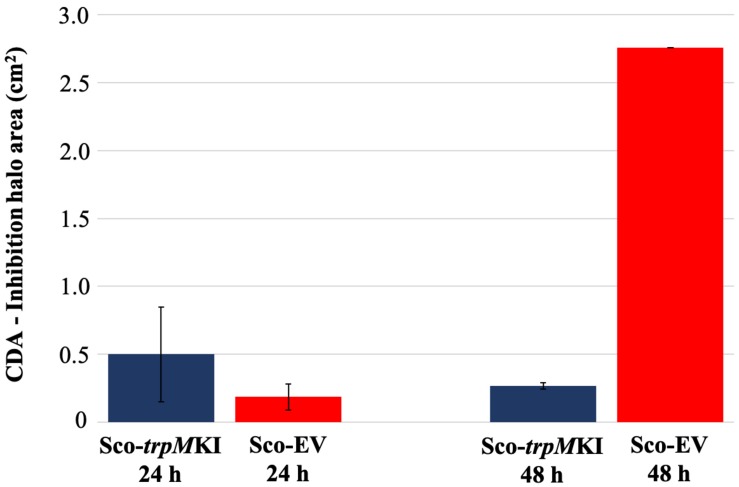
Calcium-dependent antibiotic (CDA) production in Sco-*trpM*KI and Sco-EV cultures determined by bacterial growth inhibition assay.

During solid-medium growth, Sco-*trpM*KI had a faster growth in comparison to Sco-EV since the former showed an evident substrate mycelium after 24 h, which was not present in the latter (Supplementary Figures S2A,B, respectively). After 48 h of growth, both strains showed developed hyphae with few and faint septal constrictions that were more evident in Sco-*trpM*KI (Supplementary Figures S2C,D). Spore chains were clearly visible in the 72 h-old Sco-*trpM*KI (Supplementary Figure S2E) as well as quite lagging in Sco-EV (Supplementary Figure S2F). Nonetheless, after 120 h of growth both Sco-*trp*MKI and Sco-EV had a comparable phenotype with no major morphological differences (Supplementary Figures S2G,H). This finding is in good agreement with the faster and higher biomass-yielding growth kinetics in liquid medium cultures, in which Sco-*trpM*KI had a more rapid and abundant production of biomass up to 72 h (Figure 1). Accordingly, a higher alkalinization of the growth medium was observed in Sco-*trpM*KI that was attributed to conversion of NaNO_3_ (the sole nitrogen source in the growth medium) into ammonia (Fischer et al., 2012). This suggested that Sco-*trpM*KI had an increased utilization of nitrogen consistently with its higher growth rate, further confirming that the expression of the extra copy of *trpM* influenced the growth rate accelerating morphological development progress. However, *trpM* expression did not affect the final amount of spores and undecylprodigiosin (Figure 2), but exerted a positive effect on the production of the polyketide antibiotic ACT, both in liquid and on solid media, with an amount increment ranging from 2.5 to 3 times in comparison to Sco-EV (Figure 3). On the other hand, the expression of the extra copy of *trpM* had a negative effect on CDA, with a more than fivefold decrement of CDA production compared to Sco-EV (Figure 4).

### Proteomic and Metabolic Profiles of the *trpM* Knock-in Mutant Strain

A 2D-DIGE proteomic analysis (Supplementary Figure S3) coupled with nanoLC-ESI-LIT-MS/MS was performed to infer metabolic pathways influenced by the expression of *trpM* in Sco-*trpM*KI. This investigation led to the identification of 40 differentially represented proteins, with 23 and 17 over- and down-represented ones in Sco-*trpM*KI compared to Sco-EV, respectively (Table 3, Supplementary Figure S4A and Supplementary Tables S1, S2). According to a KEGG orthology-based classification, most of them are involved in translation, carbon metabolism and folding-sorting-degradation of proteins (Table 3). Above all, the over-representation of proteins involved in protein synthesis, DNA synthesis and energy metabolism was consistent with the improved growth of Sco-*trpM*KI. On the other hand, the effect of *trpM* expression on growth was associated with a down-representation of several glycolytic enzymes as well as with the simultaneous over- and down-representation of SCO5042 (fumarate hydratase) and SCO4827 (malate dehydrogenase), respectively, which take part into tricarboxylic acid (TCA) cycle. Interestingly, the serine hydroxymethyltransferase (GlyA) involved in interconversion of serine and glycine was down-represented in the Sco-*trpM*KI strain, thus revealing that TrpM can affect the biosynthesis of serine, which is in turn a direct precursor of Trp.

**TABLE 3:** Differentially represented proteins in Sco-*trpM*KI in comparison to Sco-EV.

SCO ID	Protein description	Differential abundance^1^	*p* (ANOVA)	Functional classification^2^
SCO2179	Probable cytosol aminopeptidase	–1.31	4.42 × 10^–3^	Amino acid metabolism; Folding, sorting, and degradation

SCO4837	Serine hydroxymethyltransferase	–1.30	2.04 × 10^–4^	Amino acid metabolism

SCO1947	Glyceraldehyde-3-phosphate dehydrogenase	–1.32	1.67 × 10^–2^	Carbon metabolism
SCO3649	Fructose-bisphosphate aldolase	–1.52	1.87 × 10^–4^	
SCO3649	Fructose-bisphosphate aldolase	–1.61	1.22 × 10^–4^	
SCO4209	2,3-bisphosphoglycerate-dependent phosphoglycerate mutase	–1.37	2.01 × 10^–3^	
SCO4827	Malate dehydrogenase	–1.54	4.44 × 10^–5^	
SCO5042	Fumarate hydratase class II	2.14	1.82 × 10^–5^	

SCO2619	ATP-dependent Clp protease proteolytic subunit 1	1.70	1.77 × 10^–2^	Cell growth and death; Folding, sorting and degradation

SCO5371	ATP synthase subunit alpha	1.35	2.00 × 10^–3^	Energy metabolism
SCO5374	ATP synthase epsilon chain	1.90	1.13 × 10^–3^	

SCO1644	Proteasome subunit beta	1.75	1.62 × 10^–3^	Folding, sorting and degradation
SCO4296	60 kDa chaperonin 2	1.35	1.18 × 10^–2^	
SCO4296	60 kDa chaperonin 2	1.52	1.85 × 10^–2^	
SCO4296	60 kDa chaperonin 2	2.33	1.54 × 10^–3^	
SCO4296	60 kDa chaperonin 2	1.63	8.03 × 10^–4^	

SCO1523	Pyridoxal 5′-phosphate synthase subunit PdxS	–1.83	1.71 × 10^–3^	Metabolism of cofactors and vitamins
SCO1523	Pyridoxal 5′-phosphate synthase subunit PdxS	–2.23	9.56 × 10^–7^	
SCO1523	Pyridoxal 5′-phosphate synthase subunit PdxS	–1.31	6.82 × 10^–3^	
SCO4824	Bifunctional protein FolD	4.61	5.59 × 10^–8^	

SCO4041	Uracil phosphoribosyltransferase	2.05	2.12 × 10^–4^	Nucleotide metabolism

SCO0409	Spore-associated protein A	1.45	1.19 × 10^–2^	Other
SCO0409	Spore-associated protein A	–2.42	4.11 × 10^–4^	
SCO4636	UPF0336 protein SCO4636	–1.34	3.56 × 10^–2^	

SCO2633	Superoxide dismutase [Fe-Zn] 1	2.08	2.75 × 10^–7^	Oxidoreduction; Stress response
SCO2633	Superoxide dismutase [Fe-Zn] 1	–1.47	1.91 × 10^–3^	
SCO2633	Superoxide dismutase [Fe-Zn] 1	–1.69	7.44 × 10^–4^	

SCO3907	Single-stranded DNA-binding protein 2	1.77	9.16 × 10^–4^	Replication and repair; Stress response

SCO0527	Cold shock protein ScoF	–1.50	3.63 × 10^–2^	Transcription; Stress response

SCO1505	30S ribosomal protein S4	1.82	8.98 × 10^–5^	Translation
SCO1599	50S ribosomal protein L35	–1.59	3.90 × 10^–3^	
SCO4702	50S ribosomal protein L3	1.47	1.65 × 10^–2^	
SCO4702	50S ribosomal protein L3	–1.30	3.66 × 10^–2^	
SCO4702	50S ribosomal protein L3	2.53	3.36 × 10^–6^	
SCO4703	50S ribosomal protein L4	1.46	1.42 × 10^–3^	
SCO4711	30S ribosomal protein S17	1.53	3.01 × 10^–2^	
SCO4713	50S ribosomal protein L24	1.30	6.14 × 10^–3^	
SCO4735	30S ribosomal protein S9	1.61	1.18 × 10^–6^	
SCO5624	30S ribosomal protein S2	1.32	2.52 × 10^–3^	
SCO5624	30S ribosomal protein S2	1.96	3.94 × 10^–6^	

*^1^Positive and negative values stand for up- and down-representation in Sco-*trpM*KI, respectively. ^2^Functional classification is based on KEGG orthology.*

Notwithstanding these proteomic determinations, similar intracellular levels for serine and Trp were ascertained in Sco-*trpM*KI and Sco-EV strains by quantitative metabolite measurements (Table 4 and Supplementary Tables S3, S4). Indeed, this analysis showed a down-representation of tyrosine and phenylalanine (Table 4 and Supplementary Table S3) competing with Trp for metabolic intermediates. In general, most differentially abundant compounds were related to: (i) amino acid, (ii) carbon metabolism intermediates; (iii) nucleotide metabolism intermediates (Supplementary Figure S4B); most of these compounds were down-represented in Sco-*trpM*KI strain. Exceptions were histidine, inosine, AMP, methylmalonate and succinate, which were over-represented in the Sco-*trpM*KI in the respect of Sco-EV (Table 4 and Supplementary Table S3). This observation, together with the down-representation of compounds belonging to carbon metabolism pathways (i.e., glycolysis and TCA cycle), depicted a metabolic profile that was in good agreement with that deriving from proteomic results (Supplementary Figure S5).

**TABLE 4:** Differentially represented metabolites in Sco-*trpM*KI in comparison to Sco-EV.

Compound	Differential abundance^1^	*P* (Student’s *t*-test)	Functional classification^2^
Aspartic acid	−3.23	2.76 × 10^–2^	Amino acid metabolism
Arginine	−1.37	4.65 × 10^–2^	
Phenylalanine	−1.73	3.31 × 10^–2^	
Tyrosine	−1.53	2.45 × 10^–6^	
Histidine	NA	NA	
Glutamic acid	−1.37	3.86 × 10^–2^	
Glutamine	−1.69	1.26 × 10^–2^	

Glucosamine 6-phosphate	−2.26	2.81 × 10^–2^	Glycolysis; Pentose phosphate pathway
3-phosphoglycerate	−1.91	4.79 × 10^–2^	
Fructose 6-phosphate	−1.67	3.43 × 10^–2^	
Glucose 6-phosphate	−3.13	4.20 × 10^–3^	
6-phosphogluconate	−1.84	4.62 × 10^–2^	

2-oxoglutarate	−1.63	2.78 × 10^–2^	TCA cycle
Fumarate	NA	NA	
Citrate	−1.36	4.20 × 10^–2^	
Methylmalonate	1.70	4.91 × 10^–2^	
Succinate	1.72	4.59 × 10^–2^	
Malate	−1.90	4.21 × 10^–2^	
Oxalate	NA	NA	

AMP	NA	NA	Nucleotide metabolism
CMP	−2.97	2.33 × 10^–2^	
UMP	−3.37	4.64 × 10^–3^	
Uracil	−2.15	4.91 × 10^–2^	
GMP	NA	NA	
Inosine	2.04	4.54 × 10^–2^	
Guanosine	−1.34	1.41 × 10^–2^	

*^1^Positive and negative values stand for up- and down-representation in Sco-*trpM*KI, respectively. NA, not applicable. ^2^Functional classification is based on KEGG orthology.*

### Enzymatic Activity of PepA

Different experimental data suggested functional relations between PepA and TrpM. In particular, Palazzotto et al. (2016) identified PepA as a putative TrpM-interacting protein through pull-down experiments, and then confirmed this protein-protein interaction *in vitro* by an adenylate cyclase-based bacterial two-hybrid assay. In this work, PepA was down-represented in Sco-*trpM*KI in comparison with Sco-EV (Table 3 and Supplementary Tables S1, S2); on the contrary, a higher expression of this gene was observed by qRT-PCR (Supplementary Figure S6). This finding suggested the presence of putative post-transcriptional and/or post-translational regulatory mechanisms controlling the final representation of the active protein, as already globally described elsewhere (Jayapal et al., 2008; Jeong et al., 2016).

**FIGURE 5:**
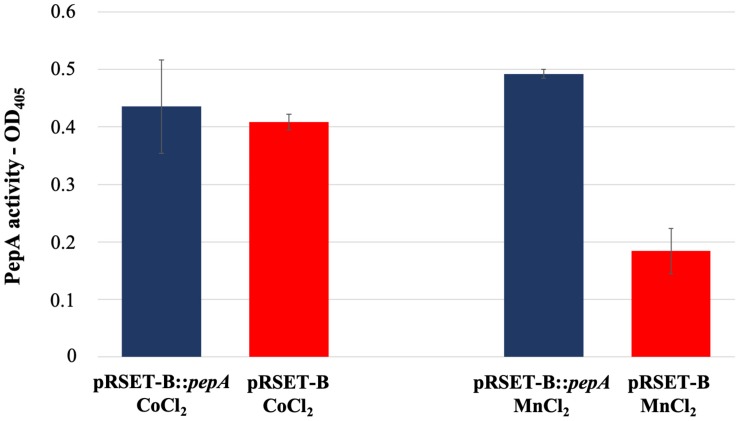
Leucine aminopeptidase activity measured in the WS protein fraction extracted from cultures of *E. coli* BL21-AI pRSET-B:*pepA* and *E. coli* BL21-AI pRSET-B.

As reported in the UniProtKB database, the protein product of *pepA* is a putative cytosol aminopeptidase (entry identifier Q9S2Q7) belonging to the peptidase M17 family. In order to have an experimental confirmation of the PepA function, *pepA* was cloned in the expression vector pRSET-B and over-expressed in *E. coli* as His-tagged protein. Purification of His-tagged PepA under native conditions was not successful, because of the low amount of the soluble protein, due to the undesired formation of inclusion bodies. Thus, WS protein fractions obtained from the *pepA*-overexpressing *E. coli* strain and from the strain carrying the vector pRSET-B were used for comparative enzymatic assay experiments, using the latter as control. In agreement with Song et al. (2013), PepA activity was not revealed in the protein extraction in presence of CoCl_2_, while the use of MnCl_2_ notably highlighted for the first time that PepA exerted the predicted aminopeptidase activity (Figure 5).

## Discussion

The results described in this work revealed that *trpM* boosts the morpho-physiological differentiation of *S. coelicolor*. This positive effect was inferred from the accumulation of biomass in liquid cultures and by the mycelium morphology on solid growth medium observed in Sco-*trpM*KI in comparison to Sco-EV. In agreement, SCO0409 product – known as spore-associated protein A or SapA – is deregulated as shown by differential proteomic analysis. This protein, along with SapB, is expressed in aerial hyphae and is localized on the external surface of spores, contributing to the shaping of spores and to their hydrophobicity (Guijarro et al., 1988; Willey et al., 1991).

The synthesis of the three major secondary metabolites of *S. coelicolor* – i.e., ACT, undecylprodigiosin and CDA – was studied in the *trpM* knock-in strain. It revealed a stimulatory effect only on ACT production. This finding is consistent with the results described by Palazzotto et al. (2016) concerning a *trpM*-knockout mutant strain, whose production of ACT was impaired upon cultivation on minimal medium. These results suggest that *trpM* expression could influence ACT biosynthesis through an indirect regulation of central carbon metabolism. Indeed, the biosynthesis of antibiotics in *S. coelicolor* is greatly affected by precursor supply. In the *trpM* knock-in strain, the relative abundances of two TCA cycle enzymes – i.e., malate dehydrogenase (SCO4827) and fumarate hydratase class II (SCO5042) – suggested a differential abundance of TCA intermediates. Accordingly, the metabolite profile analysis revealed the accumulation of succinate, which can be related also with the accumulation of methylmalonate, a metabolic intermediate of the branched-chain amino acid catabolism providing precursors for the biosynthesis of type II polyketide antibiotics, like ACT (Stirrett et al., 2009). However, the exact metabolic routes possibly leading to an increment of malonyl-CoA, which is an extending unit precursor of the ACT backbone (Revill et al., 1995; Hopwood, 1997; Rawlings, 1999) were not inferred from the proteomic and metabolic profiles of Sco-*trpM*KI strain.

On the other hand, the decrement of CDA production may be related to the altered amounts of amino acids observed in Sco-*trpM*KI strain. Indeed, the biosynthesis of CDA is dependent on amino acid availability. In particular, the peptide moiety of CDA includes residues of serine, threonine, tryptophan, glutamic acid, hydroxyphenylglycine, aspartic acid, glycine, and asparagine (Hojati et al., 2002). Although tyrosine is not part of CDA backbone directly, it is known that CDA biosynthesis intersects tyrosine metabolism since the metabolic intermediary compound 4-hydroxyphenylpyruvate – a product of tyrosine catabolism that is also involved in CDA precursor biosynthesis – exerts a positive effect on the regulatory protein HpdR. This regulator in turn controls genes required for tyrosine catabolism and CDA biosynthesis (Liu et al., 2013). In this work, both glutamic and aspartic acid, which are present in CDA backbone, were down-represented in Sco-*trpM*KI reinforcing the hypothesis that CDA yield was strongly affected by amino acid availability. However, it is noteworthy that Trp levels – along with some of its precursors anthranilate, chorismate and phenylpyruvate – seemed to be not limiting for CDA biosynthesis, since these chemical species were not differentially represented in Sco-*trpM*KI in comparison to Sco-EV.

In the *trpM* knock-in mutant, the decreased intracellular amount of tyrosine and phenylalanine – sharing different biosynthetic steps with Trp – parallels the decrement of arginine, glutamate and glutamine: these three amino acids derive from TCA cycle intermediates oxaloacetate (i.e., arginine) and alpha-ketoglutarate (i.e., glutamate and glutamine) and, moreover, biosynthesis of arginine is directly linked to glutamate/glutamine through ammonium assimilation (Lu, 2006; Lewis et al., 2011). On the other hand, the decreased amount of these amino acids is coupled with increment of histidine whose biosynthesis intersects different metabolic pathways, including those involved in Trp, purine and pyrimidine biosynthesis, as well as in C1 metabolism (Schwentner et al., 2019). In fact, Trp, purine, pyrimidine and histidine biosyntheses share the phosphoribosyl pyrophosphate (PRPP), a metabolite intermediate originating from ribose-5P. Tetrahydrofolate (THF) is a key cofactor in C1 metabolism; a metabolic source for the generation of loaded THF molecules is the reaction of serine hydroxymethyltransferase (GlyA – SCO4837) that interconverts glycine and serine. This last amino acid is a Trp precursor required in the last biosynthetic step of condensation with indole. Interestingly, a differential abundance was observed in Sco-*trpM*KI strain for GlyA and FolD (SCO4824, a bifunctional protein with methylenetetrahydrofolate dehydrogenase and methenyltetrahydrofolate cyclohydrolase activities), with the latter enzyme being required in biosynthesis of THF derivatives. Additionally, THF is involved in the synthesis of pantothenate that is a key precursor for the biosynthesis of essential cofactor coenzyme A (CoA) and carrier proteins having a phosphopantetheine prosthetic group such as polyketide synthases and non-ribosomal peptide synthetases (Leonardi and Jackowski, 2007). Altogether these results suggest that *trpM* over-expression stimulates metabolic circuits that are interconnected with each other and with Trp biosynthesis but whose regulation and functions still deserve further investigations. The alteration of amino acid biosynthetic pathways due to *trpM* over-expression could be also linked to the observed alkalinization of medium in liquid culture of Sco-*trpM*KI. Indeed, glutamate/glutamine and arginine play a key role in nitrogen assimilation (Lu, 2006; Lewis et al., 2011) and their down-representation could account for an increased amount of free ammonium causing medium alkalinization. In addition, it has been recently reported that different *Streptomyces* species, including *S. coelicolor*, are able to produce ammonia in presence of higher levels of glycine, exerting (i) an antimicrobial activity and (ii) reinforcing the efficiency of other antibiotics through perturbation of membrane permeability in target cells (Avalos et al., 2019). At this regard, it would be interesting to clarify the role of GlyA, which converts serine in glycine, and its putative interplay with TrpM.

The altered metabolism of amino acids could also indirectly contribute to increase ACT biosynthesis in two different manners. Indeed, on one hand, the augmented synthesis of some amino acids (e.g., histidine) could cause the occurrence of oxidative stress in Sco-*trpM*KI and, at this regard, it has been proposed that ACT could be a mean to reduce oxidative metabolism in *S. coelicolor* (Esnault et al., 2017). On the other hand, same authors proposed that metabolite shortage (like that of aspartic acid, arginine, phenylalanine, tyrosine, glutamic acid and glutamine in Sco-*trpM*KI) could act as a trigger as well. In particular, considering that *trpM* expression is positively correlated with ACT biosynthesis, its possible role in limiting oxidative stress through control of ACT concentration deserves further investigation: structural studies of TrpM could clarify, for example, if the two cysteine residues in position 19 and 24 are part of a 2Fe-2S cluster able to sense redox stress and induce conformational changes in TrpM structure. If this is the case, the experimental proof reported by Palazzotto et al. (2016) about the ability of TrpM to form dimers would suggest that a 2Fe-2S cluster could form upon dimerization, providing the four cysteine residues needed.

The differential proteomic analysis revealed that proteins involved in protein synthesis were more abundant in Sco-*trpM*KI strain. These ones included ribosomal proteins and chaperonins. The over-representation of GroEL (SCO4296) was in agreement with its well-documented involvement in metabolism during stationary phase, in morpho-physiological differentiation and in production of secondary metabolites, such as ACT (Kim et al., 2008). Moreover, proteins taking part in nucleotide metabolism and in replication and repair of DNA were more abundant as well, mirroring, at molecular level, the higher accumulation of biomass in Sco-*trpM*KI strain. As it is inferred from metabolite profile analysis, Trp biosynthesis does not seem dramatically altered in Sco-*trpM*KI, in opposition to the findings obtained for the *trpM*-knockout mutant strain (Palazzotto et al., 2016). Indeed, in the latter, some proteins involved in Trp biosynthesis (AroF and TrpE) or its utilization (TrpS) were more abundant, suggesting a compensatory effect probably due to Trp shortage. In Sco-*trpM*KI we did not observe up-regulation of proteins involved in Trp biosynthesis, neither an accumulation of Trp or its precursors (i.e., anthranilate and chorismite), suggesting that the *trpM* knock-in mutation does not exert dramatic effects on Trp metabolism as *trpM*-knockout mutation does. Main differences regarding these two mutant strains are reported in Table 5.

**TABLE 5:** Main differences between Sco-*trpM*KI and the *trpM*-knockout mutant strains in comparison to their corresponding control strains (Sco-EV and *S. coelicolor* M145, respectively).

Phenotype	Sco-*trpM*KI^1^	*trpM*-knockout^1^*
Growth rate	+	−
Morphological differentiation	+	−
Spore amount	=	−
ACT	+	−
RED	=	N.D.
CDA	−	N.D.
L-Trp biosynthetic enzymes	=	+
L-Trp precursors	=	N.D.

^1^+, yield increment/stimulation; −, yield decrement/inhibition; =, no difference observed; N.D., not determined. *Palazzotto et al. (2016).

In addition, the relationship between stress response proteins and streptomycete development is well known since it has been described by Puglia et al. (1995). In the *trpM* knock-in strain there was also an accumulation of stress response proteins. Analogously, a stimulatory role of Trp on *S. coelicolor* morphological and physiological differentiation was described in Palazzotto et al. (2015) and, among the proteins accumulated in the strain upon Trp supplementation into growth medium, proteins related to stress response were observed, including also oxidative stress proteins. Anyhow, it is still unclear how the up-regulation of *trpM* can exert the pleiotropic effects observed.

As reported by Palazzotto et al. (2016), TrpM interacts with PepA. In addition, qRT-PCR analysis showed that *pepA* was more expressed in Sco-*trpM*KI strain, while proteomics showed that the corresponding protein was less abundant therein. These results suggest the presence of post-transcriptional and/or post-translational regulatory mechanisms that regulate PepA levels. It would be very interesting to find out if this putative regulation is actually due to the TrpM-PepA interaction; this point deserves further investigations.

In this work, *pepA* was cloned and over-expressed in *E. coli* to study the putative enzymatic activity of the corresponding protein. Here, we demonstrated for the first time that PepA has an enzymatic activity that corresponds to the predicted one, shedding new lights on the role of TrpM-PepA interaction. It will be important to know whether or not TrpM affects PepA activity upon binding and, if this is the case, whether the effect is positive or negative. It is well documented that bacterial differentiation can be regulated through the protease-mediated selective degradation of regulators (Gottesman and Maurizi, 1992; Baker and Sauer, 2006). For instance, in *S. coelicolor*, the ATP-dependent serine proteases ClpP have a role in differentiation; in addition, protease inactivation was shown to induce both “bald” phenotype and lack of pigmentated antibiotics (i.e., ACT and undecylprodigiosin) (de Creìcy-Lagard et al., 1999; Bellier and Mazodier, 2004). Although PepA does not belong to the same protease class of ClpP proteins, it is interesting to note that its inactivation caused an enhancement of sporulation and production of ACT, thus suggesting a key role in *S. coelicolor* differentiation (Song et al., 2013; Xu et al., 2019).

Many genes involved in diverse cellular processes different from ACT production – such as metabolism of amino acids, carbohydrate, cell wall and DNA – or having unknown functions were shown to affect ACT production and/or actinomycete development (Gehring et al., 2004; Xu et al., 2019). In this work, TrpM was shown to play a role in regulating *S. coelicolor* growth and differentiation, specifically stimulating ACT production. In particular, our experimental data highlighted the stimulation of a regulatory circuit involving amino acid, cofactor and protein biosynthesis. In this circuit, a possible role could be exerted by the interplay between TrpM and the aminopeptidase PepA. Further studies are necessary to deeply understand the specific molecular mode of action of TrpM, explaining if and how TrpM-PepA interaction has a direct influence on the growth, antibiotic biosynthesis and developmental program of *S. coelicolor*. To such purpose, it will be important to determine the temporal expression profile of both TrpM and PepA and to identify cellular targets of the aminopeptidase activity of PepA, also considering that the augmented expression of *trpM* and the inactivation of *pepA* (Song et al., 2013) both positively influence ACT production in *S. coelicolor*. Furthermore, even the putative interaction between TrpM and the ribosomal protein S2 (SCO5624) will deserve a specific investigation: in fact, it was previously identified as a putative interacting protein of TrpM (Palazzotto et al., 2016) and it was interestingly over-represented in Sco-*trpM*KI as well. Finally, considering that TrpM is conserved among *Streptomyces* species, it should be investigated whether it is able to exert a positive effect even on other secondary metabolite biosynthesis, especially polyketides. Indeed, this chemical class includes several compounds with antibiotic, antitumoral and immunosuppressant activity. So, the development of recombinant strains with altered expression of TrpM and/or PepA could be promising to enhance industrial production of these molecules.

## Data Availability Statement

Proteomic data have been submitted to the PRIDE database under the project accession PXD015937.

## Author Contributions

AV, EP, GR, LB, and TF performed the experiments. AS and GG supervised the experiments and analyzed the results. AV, AP, and GG designed the experiments. AP and GG conceived the project. AV wrote the manuscript with the help of AS and GG. All authors read and approved the manuscript.

## Conflict of Interest

The authors declare that the research was conducted in the absence of any commercial or financial relationships that could be construed as a potential conflict of interest.

## Supplementary Material

FIGURE S1 qRT-PCR analysis showing the relative quantification (RQ) of *trpM* expression in Sco-*trpM*KI and Sco-EV.

FIGURE S2 SEM images of Sco-*trpM*KI and Sco-EV after 24, 48, 72, and 120 h of growth on solid MM. **(A)** Sco-*trpM*KI 24 h. **(B)** Sco-EV 24 h. **(C)** Sco-*trpM*KI 48 h. **(D)** Sco-EV 48 h. **(E)** Sco-*trpM*KI 72 h. **(F)** Sco-EV 72 h. **(G)** Sco-*trpM*KI 120 h. **(H)** Sco-EV 120 h.

FIGURE S3 Representative 2D-proteome map of whole protein extracts obtained from Sco-*trpM*KI. Differentially represented proteins listed in Supplementary Tables S1, S2 are labeled.

FIGURE S4 Distribution into functional classes of differentially represented proteins **(A)** and metabolites **(B)** when Sco-*trpM*KI and Sco-EV strains were compared. Percentages indicate the relative abundance of each functional class.

FIGURE S5 Metabolic pathway map showing over-represented proteins (red), down-represented proteins (light green), over-represented metabolites (maroon), and down-represented metabolites (dark green) in Sco-*trpM*KI in comparison to Sco-EV. Only proteins involved in the labeled metabolic pathways are reported. Proteins are indicated with the corresponding genomic locus (i.e., SCOXXXX), while metabolites with their KEGG identifier (i.e., CXXXXX). This map was generated using the web-based tool Interactive Pathways Explorer v3 (iPath3) (Darzi et al., 2018).

FIGURE S6 qRT-PCR analysis showing relative quantification (RQ) of *pepA* expression in Sco-*trpM*KI and Sco-EV.

TABLE S1 Description, abundance profile, functional classification and mass spectrometry identification parameters of differentially represented proteins. Functional classification is based on KEGG orthology.

TABLE S2 Protein identification details of spots observed as differentially represented.

TABLE S3 Description, abundance profile and functional classification of differentially represented metabolites. Functional classification is based on KEGG orthology.

TABLE S4 Description, abundance profile and functional classification of not differentially represented metabolites. Functional classification is based on KEGG orthology.
