# Supplementary figures and images for: The Streptomyces coelicolor Small ORF trpM Stimulates Growth and Morphological Development and Exerts Opposite Effects on Actinorhodin and Calcium-Dependent Antibiotic Production
Source: Front Microbiol. 2020 Feb 19;11:224. doi: 10.3389/fmicb.2020.00224 (PMC7042404; doi:10.3389/fmicb.2020.00224)

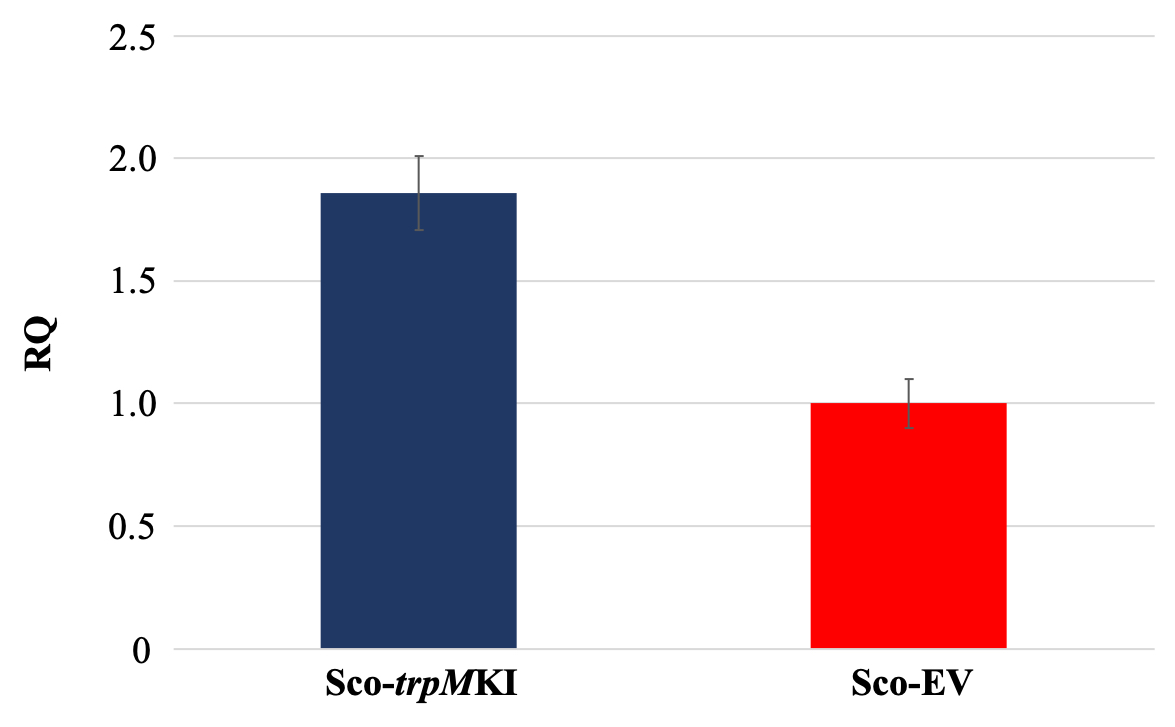

Supplement: FIGURE S1 — qRT-PCR analysis showing the relative quantification (RQ) of trpM expression in Sco-trpMKI and Sco-EV. [file Image_1.JPEG]

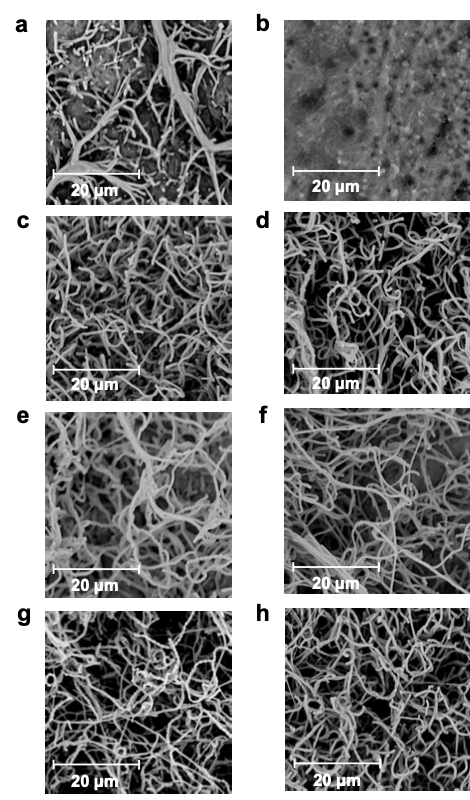

Supplement: FIGURE S2 — SEM images of Sco-trpMKI and Sco-EV after 24, 48, 72, and 120 h of growth on solid MM. (A) Sco-trpMKI 24 h. (B) Sco-EV 24 h. (C) Sco-trpMKI 48 h. (D) Sco-EV 48 h. (E) Sco-trpMKI 72 h. (F) Sco-EV 72 h. (G) Sco-trpMKI 120 h. (H) Sco-EV 120 h. [file Image_2.JPEG]

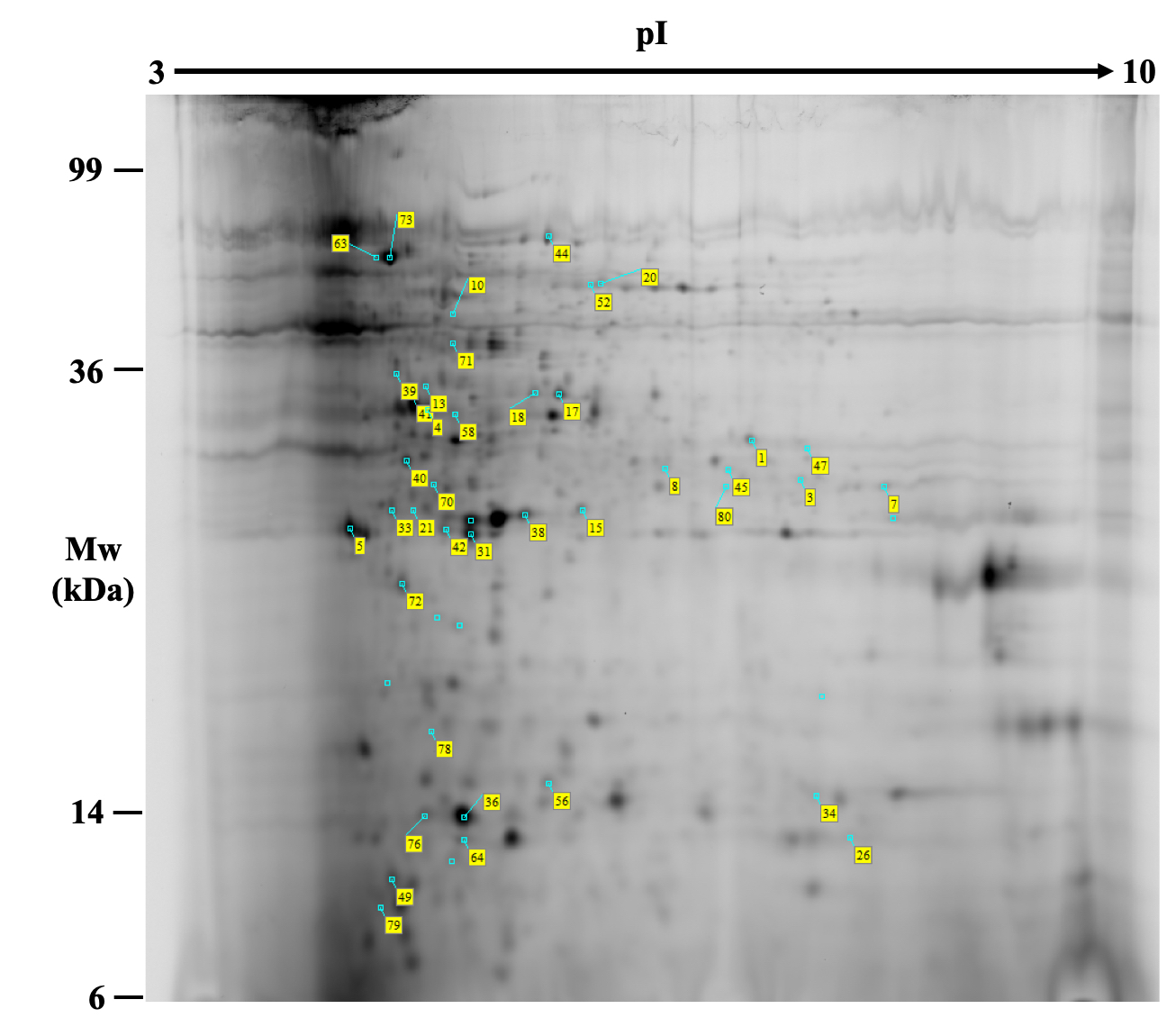

Supplement: FIGURE S3 — Representative 2D-proteome map of whole protein extracts obtained from Sco-trpMKI. Differentially represented proteins listed in Supplementary Tables S1, S2 are labeled. [file Image_3.JPEG]

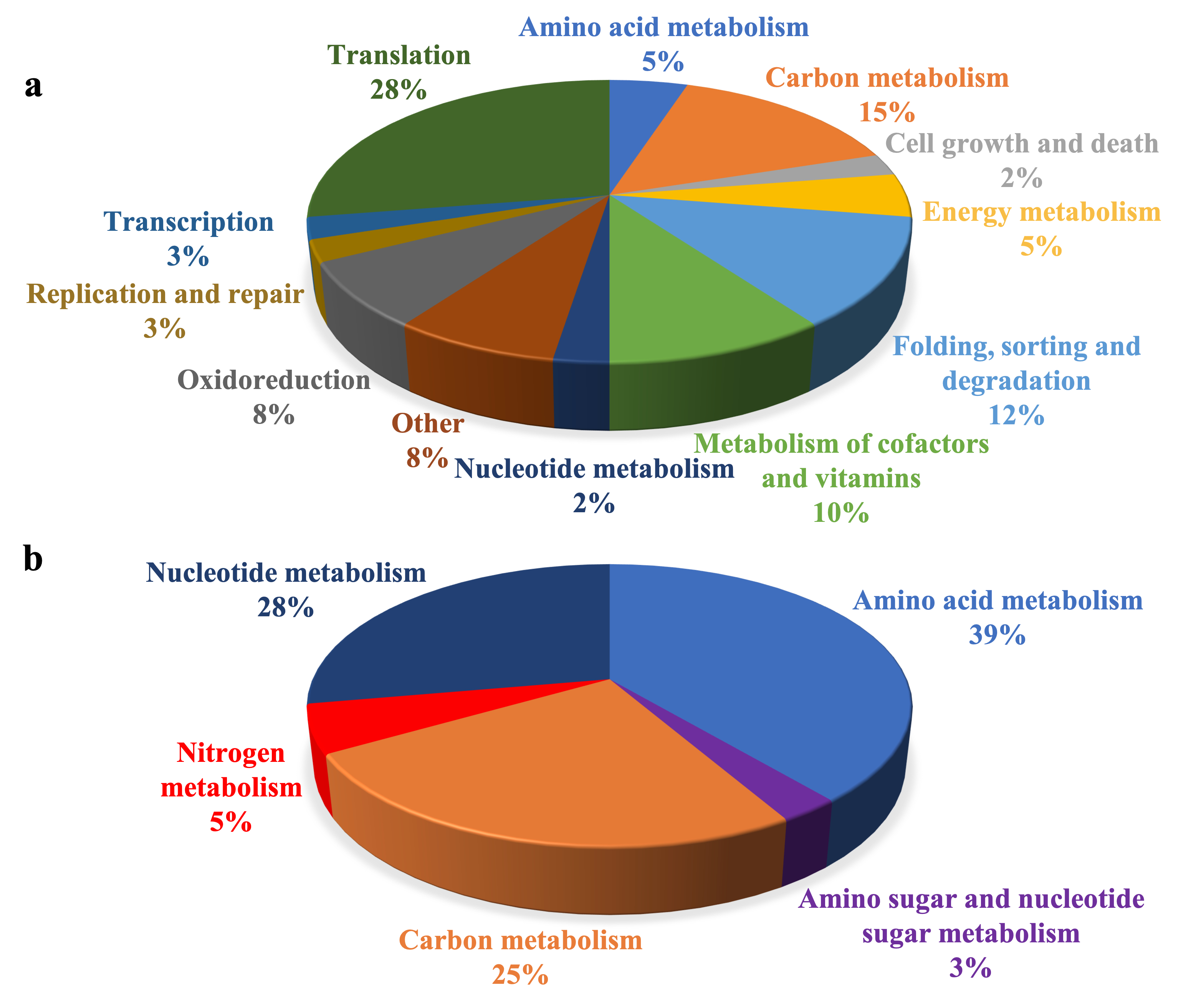

Supplement: FIGURE S4 — Distribution into functional classes of differentially represented proteins (A) and metabolites (B) when Sco-trpMKI and Sco-EV strains were compared. Percentages indicate the relative abundance of each functional class. [file Image_4.JPEG]

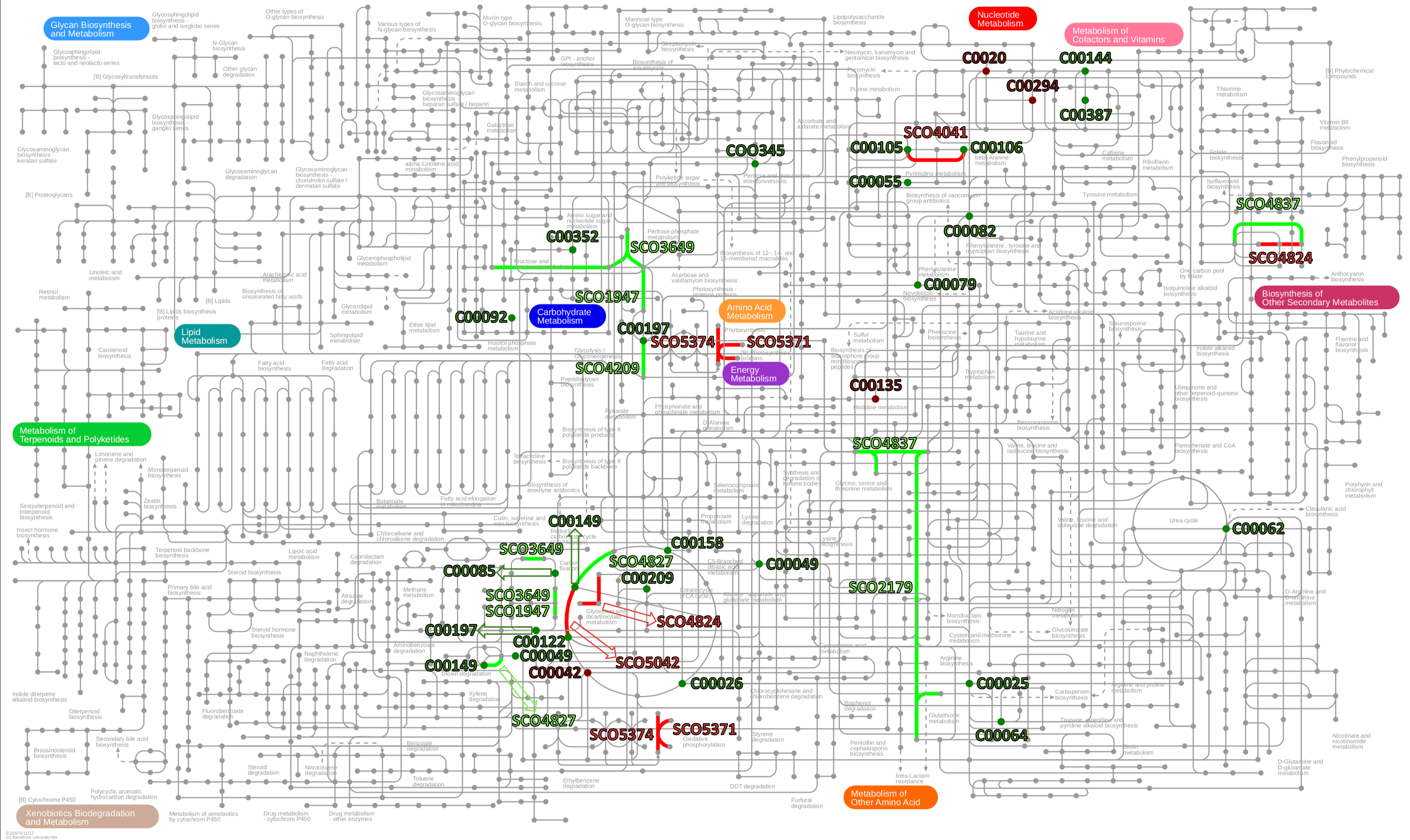

Supplement: FIGURE S5 — Metabolic pathway map showing over-represented proteins (red), down-represented proteins (light green), over-represented metabolites (maroon), and down-represented metabolites (dark green) in Sco-trpMKI in comparison to Sco-EV. Only proteins involved in the labeled metabolic pathways are reported. Proteins are indicated with the corresponding genomic locus (i.e., SCOXXXX), while metabolites with their KEGG identifier (i.e., CXXXXX). This map was generated using the web-based tool Interactive Pathways Explorer v3 (iPath3) (Darzi et al., 2018). [file Image_5.JPEG]

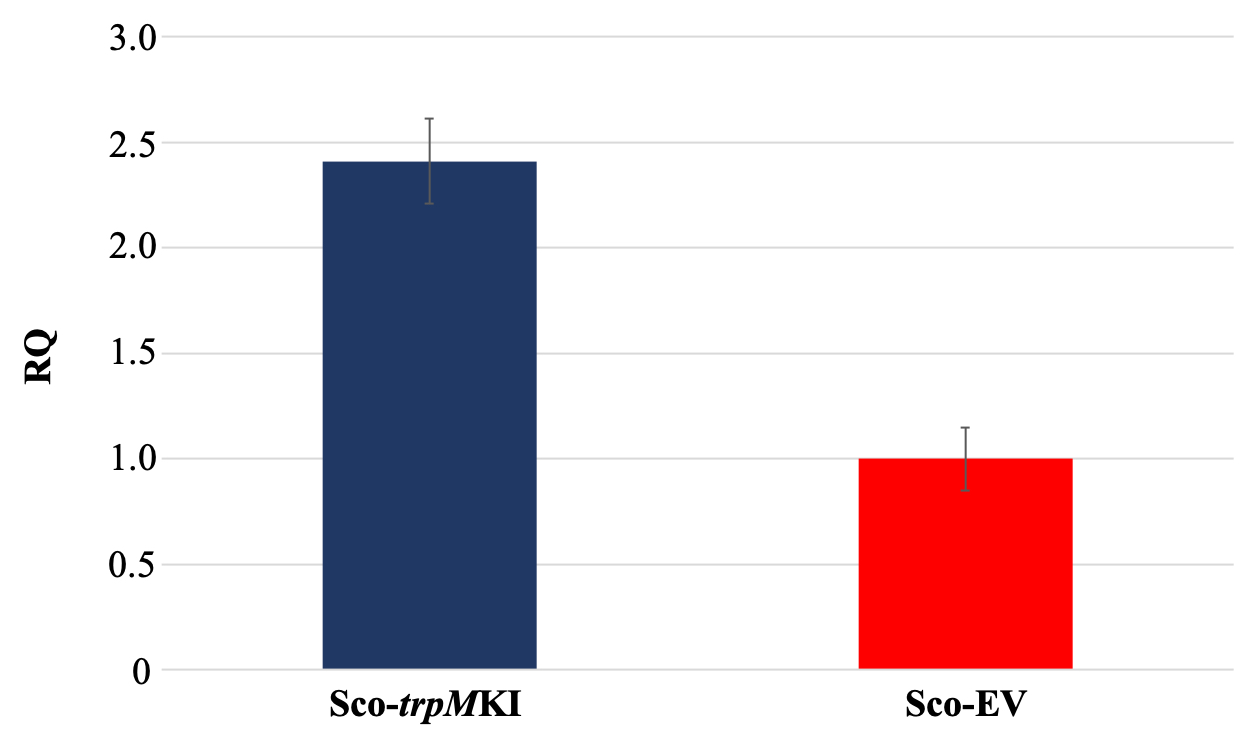

Supplement: FIGURE S6 — qRT-PCR analysis showing relative quantification (RQ) of pepA expression in Sco-trpMKI and Sco-EV. [file Image_6.JPEG]
